# Risk Factors for Attempted Suicide and Suicide Death Among South-East Asian Women: A Scoping Review

**DOI:** 10.3390/ijerph21121658

**Published:** 2024-12-12

**Authors:** Anil Fastenau, Matthew Willis, Srilekha Penna, Lahari Yaddanapudi, Madhumitha Balaji, Rahul Shidhaye, Eva Pilot

**Affiliations:** 1 Marie Adelaide Leprosy Center, Karachi 74400, Pakistan; anil.fastenau@dahw.de; 2 German Leprosy and Tuberculosis Relief Association (DAHW), 97080 Wuerzburg, Germany; srilekha.penna@glraindia.in; 3 Heidelberg Institute of Global Health, University of Heidelberg, 69120 Heidelberg, Germany; 4 Department of Global Health, Institute of Public Health and Nursing Research, University of Bremen, 28359 Bremen, Germany; 5 Department of Health, Ethics & Society, Care and Public Health Research Institute CAPHRI, Faculty of Health, Medicine and Life Sciences, Maastricht University, 6229 ER Maastricht, The Netherlands; lahari.yaddanapudi@kit.edu (L.Y.); madhumitha.balaji@sangath.in (M.B.); r.shidhaye@maastrichtuniversity.nl (R.S.); eva.pilot@maastrichtuniversity.nl (E.P.); 6 School of Medicine, Dentistry & Biomedical Sciences, Queen’s University Belfast, Belfast BT7 1NN, UK; 7 Institute for Technology Assessment and Systems Analysis (ITAS), Karlsruhe Institute of Technology (KIT), Karlstrasse 11, 76133 Karlsruhe, Germany; 8 Sangath, Porvorim 403501, India; 9 Pravara Institute of Medical Sciences, Loni, Maharastra 413736, India; 10 Centre of Studies in Geography and Spatial Planning (CEGOT), University of Coimbra, 3004-531 Coimbra, Portugal

**Keywords:** suicide, attempted suicide, South-East Asia Region, women, risk factors, predictors

## Abstract

Worldwide, attempted suicide and suicide death are one of the leading causes of morbidity and mortality. Women in South-East Asia are especially vulnerable, as almost 50% of all global female suicides occur in the 11 countries of the WHO South-East Asia Region. This scoping literature analysis aimed to identify and analyze the predictors or risk factors for attempted suicide and suicide death among South-East Asian women. A scoping literature review was conducted. Five databases—PubMed, MEDLINE, EBSCOhost, PsycINFO, and EMBASE—were searched. Forty studies and twelve literature reviews were eligible for inclusion. Women in South-East Asia, particularly those who are young and married, living in poverty, with low or no education, living in rural areas, with no employment outside the home, with lower socioeconomic position, and living within joint families are highly vulnerable to suicidality. This review identified gender disadvantage, infertility, domestic abuse, intimate partner violence, family conflicts, husband’s alcohol misuse, child marriage, forced marriages, and dowry disputes as the most significant predictors of attempted suicide and suicide death among South-East Asian women. A better understanding of the phenomenon is essential to develop effective gender-specific and culturally appropriate suicide prevention strategies or interventions.

## 1. Introduction

According to the World Health Organization (WHO), approximately 800,000 lives are lost annually due to suicide, and the cases of attempted suicide are even higher [1]. Attempted suicides must be taken into consideration as a prior suicide attempt is a well-identified and robust risk factor for death by suicide [2]. Worldwide, suicide is responsible for half of the violent deaths in men and over 70% in women [1]. Although attempted suicide and suicide death are a global health phenomenon impacting almost all regions and populations, as much as 78% of global suicides occur in low- and middle-income countries (LMICs) [3]. Furthermore, the average suicide rate is comparatively much higher in Asia than in other parts of the world [1]. Within Asia, the WHO South-East Asia Region (SEAR) (Bangladesh, Bhutan, Democratic People’s Republic of Korea, India, Indonesia, Maldives, Myanmar, Nepal, Sri Lanka, Cambodia, Thailand, Timor-Leste) is particularly affected and represents 39% of the global suicides [4].

Although the burden of suicide morbidity and mortality in the WHO SEAR is exceptionally high, evidence of the association between suicide and its risk factors is limited [4]. In many LMICs, women are particularly vulnerable to suicide, as they are affected by poverty, potentially leading to domestic abuse from husbands, sex trafficking, limited educational opportunities, fewer job prospects, and hopelessness [5]. A broad spectrum of biological, personal, social, cultural, and economic factors can influence attempted suicide and suicide deaths [6]. On the one hand, many researchers have identified common mental disorders, poverty, recent hunger, and physical illness as the most common reasons for suicide worldwide [7]. On the other hand, there is increasing evidence in the literature that specific sociocultural factors such as gender disadvantage, domestic violence, illegitimate pregnancy, pressure to bring a large dowry, the stigma for failing to produce a son, infertility, extra-marital affairs, underage marriage, and forced marriage contribute to the risk for attempted suicide and suicide death among the women in South-East Asia [8,9]. The fragmentation and lack of research that explicitly and comprehensively identify specific suicide-related risk factors or predictors for women in the WHO SEAR demonstrates the need to map the evidence to highlight research gaps and contribute to effective suicide prevention strategies [4]. The paper’s main objective is to identify and analyze significant predictors of attempted suicide and suicide death for women in South-East Asia.

## 2. Materials and Methods

### 2.1. Search Strategy

#### Identification of Articles

Published studies, reports, and reviews were searched through a search using a comprehensive search strategy (Appendix A). Attempted suicides must also be taken into consideration, as a prior suicide attempt is a well-identified and robust risk factor for death by suicide [3]. PubMed, MEDLINE, EBSCOhost, PsycINFO, and EMBASE were searched to identify the relevant peer-reviewed and individual publications. These abstracts were examined and the full text of papers that reported on the predictors or risk factors of attempted suicide or suicide death among women in the countries of South-East Asia were retrieved. To identify additional articles, a manual search was performed, based on the bibliographies of the included studies (“snowballing”) on risk factors for female suicide.

### 2.2. Study Design

The scoping review was carried out according to the Preferred Reporting Items for Systematic Reviews and Meta-Analyses (PRISMA) guidelines for scoping reviews. The PRISMA checklist can be seen in Appendix B [10].

### 2.3. Selection Criteria

#### Eligibility, Inclusion, and Exclusion

Articles dealing with suicide deaths and suicide attempts among women in South-East Asia were included, as they were considered equally relevant for the analysis. Only the articles published, or reports issued between 1990 and May 2021, and reporting on at least one of the 11 countries of the WHO South-East Asian Region were included in this review. This timeframe was chosen to provide a contemporary overview of suicide-related risk factors among women in South-East Asia. Papers published in languages other than English were excluded, as well as book chapters, conference proceedings, dissertations, editorials, and commentaries. Furthermore, publications that only reported the prevalence of attempted suicide and suicide death among women in South-East Asia were excluded. After the initial search, the papers were screened and cross-checked by AF and LY to find out if there were any duplicates. Eventually, the full text of the selected publications was critically reviewed and assessed for eligibility by AF and LY. During the selection process, sample size, methodology, and quality of the studies were not considered as exclusion criteria.

### 2.4. Data Extraction and Analysis

All selected publications were examined again and entered into a pre-defined spreadsheet. The format of the spreadsheet included details on the title and year of publication, name of authors, risk factors, or predictors mentioned in each article specifically for women and the relevant countries. The detailed overview of all the papers selected and the identified risk factors for female suicidality by each publication are reported elsewhere (see Appendix A). For the purposes of this review, risk factors for attempted suicide and suicide death were not differentiated. Finally, a comprehensive analysis of the literature was conducted with a focus on the research questions mentioned earlier.

## 3. Results

Risk factors for suicide or attempted suicide are presented under three major categories (i) demographic and socioeconomic factors, (ii) psychosocial and cultural factors, and (iii) mental and physical health factors.

### 3.1. Study Characteristics

**Figure 1:**
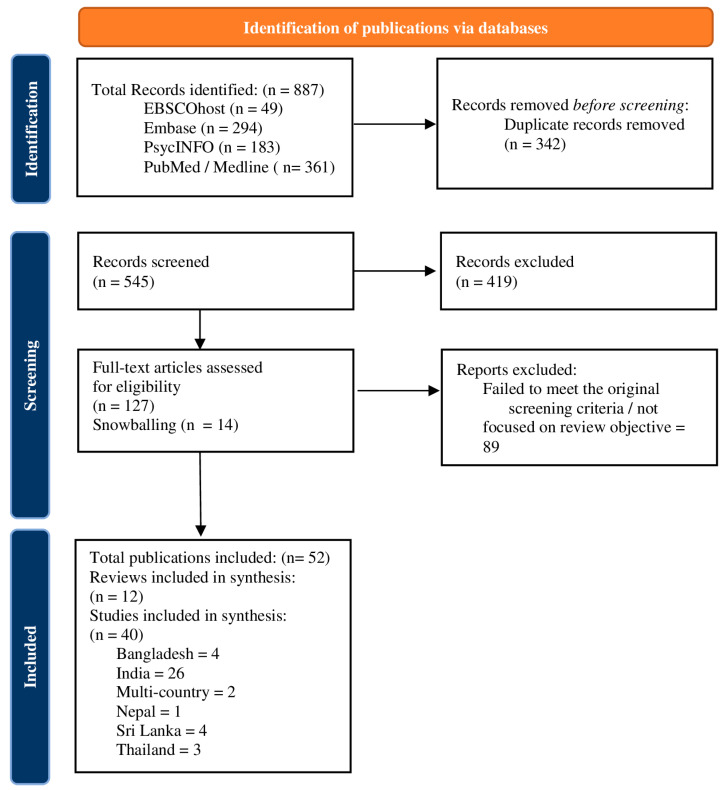
Summary of selection process for papers.

The initial database search identified 887 articles out of which 342 duplicate entries were removed and the title and abstract screening was performed for the remaining 545 articles. Full texts of 127 articles were assessed. Additionally, 14 relevant publications were identified with the snowballing technique. Finally, 40 studies and 12 literature reviews were eligible for inclusion in this analysis. The selection process for the articles is depicted in the flow chart (Figure 1).

The 52 relevant publications mainly included data from only 5 of the 11 countries in the region: Bangladesh (n = 6), India (n = 28), Nepal (n = 4), Sri Lanka (n = 5), and Thailand (n = 3). Six multi-country studies were also included. No studies from Bhutan, Democratic People’s Republic of Korea, Indonesia, Maldives, Myanmar, or Timor-Leste were found to be eligible. The papers reviewed are listed with reference number, first author’s name, publication year, title, and country or region of focus in Table S1.

### 3.2. Demographic and Socioeconomic Factors

#### 3.2.1. Age

Suicide rates for women in South-East Asia vary substantially across the age span. Being below 30 years old has been recognized as a key predictor of suicide among women in many studies from Bangladesh, India, Nepal, and Sri Lanka [9,11,12,13,14,15,16,17,18,19,20,21,22,23,24,25,26,27]. Suicide was the leading cause of mortality among Nepalese women of reproductive age (defined by the WHO as 15–49) [14,15]. Evidence from India revealed that females are at a considerably higher risk of suicide than males among younger people [9,11,13,24,28], and 60 to 70% of the women who committed suicide were younger than 25 years [11,26].

#### 3.2.2. Marital Status

The evidence for differences in female suicide risk based on marital status was inconsistent. Most studies found that married women were more likely to commit suicide [8,9,15,16,19,23,27,28,29,30,31,32,33], but three studies found that unmarried, separated, divorced, or widowed women were more likely to commit suicide [17,22,34]. In India, married women comprise the majority of suicide attempters [23,28,31,32]. In Nepal and Bangladesh, married women had a higher prevalence of overall suicidality [22,29,33,35]. However, multivariate analysis in a case-control study from Bangladesh indicated that suicide was more frequent among unmarried, divorced, and widowed women than among married women [17]. According to a population-based analysis of suicidality and its correlates in India, women who were widowed, separated, or divorced had a higher risk of suicide than those who never married [34].

#### 3.2.3. Education

A consistent association between low or no education and suicide in women was observed. Several studies found that women in Southeast Asia who were uneducated or had low educational levels were more likely to commit suicide [19,20,22,24,29,30,31,34,36,37,38,39,40]. Suicide was more prevalent among women with lower educational attainment in India than among females with higher educational attainment [22,23,24,31,34,36,40].

#### 3.2.4. Occupation

In Bangladesh and India, suicide was found to be much more frequent among housewives as compared to the women who were employed [19,27,32]. Lack of economic independence among Bangladeshi women increased their vulnerability to suicidal behavior [19,22]. Several studies found that unemployment was associated with an increased risk of suicide for Indian women [24,25,31,34,36]. In contrast to this, a cross-sectional study in rural Sri Lanka identified that women working as daily wage laborers (i.e., insecure low-income jobs) were at an increased risk of attempted suicide [37].

#### 3.2.5. Area of Residence

In Bangladesh, most cases of attempted suicide and suicide death happened in rural areas [20]. Some studies reported high rates of suicide among women from rural areas in India [31,40], but two studies from India found that females residing in urban metropolitan cities had a higher risk for suicide [32,34]. Several studies from India, Nepal, and Sri Lanka emphasized the role of easy access to pesticides for attempted suicide and suicide deaths among women, especially in rural areas [12,14,15,21,25,29].

#### 3.2.6. Poverty and Socioeconomic Position

The literature provided strong evidence of the relationship between poverty and suicidal behavior in South-East Asian women [8,14,17,19,20,23,24,26,27,29,33,34,35,36,39,41]. Likewise, lower socioeconomic position was associated with an increased risk of attempted suicide and suicide death among women in Bangladesh, India, Nepal, and Sri Lanka [13,19,20,27,33,34,35,37,39,42,43]. In Nepal, significant financial stress, low socioeconomic status, and desperate economic situations were responsible for high female suicidality [14,29,39]. Sudden unanticipated economic bankruptcy, financial crisis, and economic difficulties within the family played an important role in the elevated risk of suicide for women in India [23,24,25,26,36,41,44].

### 3.3. Marital and Familial Issues

Psychosocial and cultural factors such as gender disadvantage, intimate partner violence, domestic violence, interpersonal and family conflicts, and dowry disputes play an important role in female suicidal behavior [8,9,11,12,13,14,15,16,17,18,19,20,21,23,29,30,31,35,36,38,39,40,41,42,45,46,47,48,49,50,51,52,53,54,55]. The prevalence of further gender-specific risk factors such as alcohol use disorder in husbands, sexual abuse, childlessness, and forced marriage was reported frequently as a predictor of attempted suicide and suicide death for South-East Asian women [9,12,13,14,17,18,19,21,23,29,30,35,38,41,45,47,49,51,53,55,56]. In India, Nepal, and Sri Lanka, educational stress and academic failure were identified as independent risk factors for female suicide [21,25,29,30,44,57].

#### 3.3.1. Gender Disadvantage

Several studies from Bangladesh, India, Nepal, Sri Lanka, and Thailand identified gender disadvantage and gender-based violence as the most significant predictors of attempted suicide and suicide death among women [12,14,20,30,31,34,35,38,45,46,51]. Child marriage, adolescent pregnancy, limited agency, and lower female autonomy were identified as gender-specific and culture-specific risk factors for female suicidality in this region [19,20,30,52]. The reviewed literature also recognized forced and unacceptable marriages as culture-specific risk factors for suicide related to gender disadvantage among South Asian women [9,12,14,17,19,20,21,23,29]. Regarding other measures of gender disadvantage, the pressure on women to bear children soon after marriage and the inability of women to have children were highly associated with suicide [9,12,17,20,23,30,38,41,46,57]. Gender-segregated analysis showed that premarital sex was associated with suicidal behavior only among females in India [40]. In Bangladesh and Nepal, sex trafficking was also related to attempted suicide among women [14,20].

#### 3.3.2. Domestic Abuse and Intimate Partner Violence

The majority of the studies reported domestic violence as an independent risk factor for suicide among South-East Asian women [8,9,12,13,14,18,20,21,23,29,30,36,38,39,41,44,46,47,48,50,51,55,58]. Likewise, most studies identified sexual abuse specifically as a strong predictor of suicide among women in this region [9,20,21,30,33,38,45,49,51,53,55]. Several studies also recognized physical, sexual, and verbal intimate partner violence as a significant contributor to female suicidality throughout South-East Asia [9,17,20,21,30,38,39,44,45,49,50,51,55].

#### 3.3.3. Dowry Disputes

Dispute over bridal dowry was reported as an important factor of attempted suicide and suicide death by several studies from India [9,11,12,23,42,46,48,50,59]. Many studies found that dowry disputes were associated with an increased risk of suicide among Indian women [9,11,12,23,42,46,48,50,59]. These studies suggested that when dowry expectations are not met, the young brides are often harassed by their husbands or in-laws to the point where they are compelled to commit suicide [9,11,12,42,46,48,50,59]. In Bangladesh, dowry-based violence was identified as a frequent causative factor for female suicide [20].

#### 3.3.4. Family Conflicts and Husband’s Alcohol Misuse

Many studies from the region acknowledged that marital and family conflicts (especially with in-laws) were the major reason for suicide [14,15,16,17,18,19,20,21,22,23,24,25,27,29,30,33,36,39,41,44,46,50,54,57]. In Bangladesh, discord with spouse, marital disharmony, and emotional stress due to family quarrels were the most common risk factors associated with female suicidality [19,20,27,33]. Domestic quarrels were the main reason for attempted and suicide deaths among Indian women in 40% to 60% of the cases [22,23,25]. Extramarital sexual relationship of the husband was identified as a causative factor of female suicide by two Indian studies [36,46]. Husbands’ harmful use of alcohol was highly related to the increased risk of attempted suicide among women in Nepal, India, and Sri Lanka [12,13,18,21,29,35,41,47,56]. Some studies have highlighted the complex interconnection of family conflicts, male alcoholism, domestic violence, and female suicide [13,21,41,44,47,50].

### 3.4. Mental and Physical Health-Related Factors

A significant relationship between mental illness and female suicidal behavior was reported in many studies from South-East Asia [8,9,13,14,15,21,22,23,29,31,33,34,36,40,41,44,54,60]. However, the prevalence of mental disorders among women who attempted or committed suicide varied enormously in the literature. Generally, the data showed a rather low prevalence of mental illness among women with suicidal behavior, suggesting that other risk factors might play a greater role in female suicidality in the South-East Asian Region [8,9,13,14,15,21,23,24,27,28,29,31,34,36,40,41,54,57,60]. In Nepal, the presence of mental health problems such as anxiety, depression, and post-traumatic stress disorder was associated with increased female suicidality [14,15,29,39]. The literature from Bangladesh, India, Nepal, and Sri Lanka indicated that family history of suicidal death and prior suicide attempts were strong independent risk factors for suicide among women [15,19,20,21,25,27,33,39,44,57]. The presence of common mental disorders among Indian women increased their risk of suicide [8,22,23,25,31,32,34,36,40,41,44,54,57]. A considerable amount of evidence in the literature highlighted that among all psychiatric disorders, depression was the most common predictor for suicide in women [9,15,21,22,25,31,32,34,41,54,60]. Women with severe depressive symptoms in the post-partum period were identified as a highly vulnerable group for suicide by two different studies from India [9,60].

Long-term physical illness was identified as an independent risk factor for attempted suicide and suicide death among South-East Asian women [8,19,21,22,24,27,29,33,36,44,46]. A number of studies from India found that chronic physical illness and suffering from idiopathic pain were important reasons for attempting or committing suicide in women [8,22,23,24,36,44,46].

## 4. Discussion

In this review, we attempt to understand a complex phenomenon like suicide in an extremely diverse region of South-East Asia through studies and reviews of varying types, strengths, and limitations. The findings of this study indicate that women in South-East Asia, particularly young and married women, having low or no education, living in rural areas, having no employment outside the home, having lower socioeconomic position, living within joint families, and suffering from poverty are highly vulnerable to suicidality. During the analysis, there was sufficient and strong evidence to conclude that psychosocial and cultural factors play a predominant role in female suicidality in this region. The current manuscript identifies gender disadvantage, infertility, domestic abuse, intimate partner violence, family conflicts, husband’s alcohol misuse, child marriage, forced marriages, and dowry disputes as the most significant predictors of suicide among women in the WHO SEAR. This review also found that chronic physical illness, prior attempted suicide, and mental illness, especially depression and post-partum psychosis are important risk factors for suicide among South-East Asian women.

The publications considered for this review identify marital and family conflicts as significant predictors of female attempted suicide and suicide. These findings support the results of several other previous studies from the region, which also mention family conflict as a main predisposing factor for suicide among women [61,62,63,64,65,66,67]. The Indian National Crime Record Bureau verifies that in almost 23% of the registered cases, family conflict is the major reason leading to suicide among women below 30 years [62]. Several social and cultural factors can cause marital and family conflicts. In Bangladesh, India, and Nepal, a male child is highly preferred and there is huge pressure on women to give birth to male offspring. Giving birth to a female child could be a reason for conflict with the husband and in-laws. Likewise, female infertility could also be a cause of constant intra-household disputes and may contribute to female suicidality. Our findings highlight the association between childlessness and increased risk of suicide among South-East Asian women. This association could be mediated through family disputes leading to marital disharmony, harassment, domestic violence, reduced self-esteem, lower social status, and mental health issues. The evidence in this manuscript about the relationship between childlessness and female suicide is consistent with previous research from other developing countries [62,68,69]. Extramarital sexual relationships of the husband may also contribute to family disputes and elevated risk of suicide among wives.

Many of the interpersonal problems and conflicts in South-East Asia are often associated with culturally defined relationships, especially family and in-law relationships within the context of joint family households [41]. In contrast to the Western literature on female suicidality, the findings of this review demonstrate that living within a joint family system increases female vulnerability to suicide. Previous studies from Western authors assume that living alone is a risk factor and residing within joint family households is a protective factor [70].

Consistent with the findings of other researchers, the current review shows that the distinctive tradition of dowry is another significant precipitating factor for female vulnerability to suicide in Bangladesh and India [69,71]. The tradition of dowry consists of an ongoing series of gifts to the groom before, during, and after the marriage. The dowry disputes are a distinctive form of abuse mostly within Indian society and might force young brides to commit suicide [9,59].

Common mental disorders emerged as strong predictors of female suicidal behavior in this manuscript, as also found in previous studies [72,73,74,75]. However, in South-East Asian countries, the role of mental disorders in female suicide was not as significant as is demonstrated in the literature from high-income countries [76]. Several studies from high-income countries claim that mental illness is diagnosed in almost 90% of cases of suicide deaths and mental health issues contribute 47% to 74% of the attributable risk of suicidality [26,77]. In contrast, the findings of this review support the evidence from prior research in the WHO South-East Asia Region and highlight that psychosocial, sociocultural, and socioeconomic factors contribute a much greater or at least, an equally important, role in female suicidality in South-East Asia as mental illness [78,79]. According to the Indian National Crime Record Bureau, mental illness was the main cause of suicide only in 5% of the suicide deaths among women [62]. This review suggests that suicide among South-East Asian women is less closely associated with mental disorders and is strongly associated with psychosocial and cultural stressors interconnected with gender disadvantage experienced by women in this region.

The findings of this review clearly highlight that girls and women in South-East Asia are at increased risk of attempted suicide and suicide death. Especially, young women below the age of 30 are at a high risk of suicide. These findings are also supported by several other studies and reviews from the region [80,81,82,83,84]. Patel et al. reaffirm these findings by showing that nearly 56% of female suicides and only 40% of male suicides in India are committed at the age of below 30 years [80]. Mishra and colleagues also provide evidence that among young adults in Nepal who commit suicide, more than 70% are women [85].

In contrast with several previous studies from high-income countries (HICs), the majority of the studies reviewed show that married women in South-East Asia are a high-risk group for suicidality. A number of previous studies from developed countries, especially from Europe and the United States, have indicated that women who are single (never married, separated, divorced, or widowed) are at increased risk of suicide [86,87,88,89]. Furthermore, these studies identified being married as a protective factor for female suicidality. However, in developing countries, the evidence is insufficient to conclude that being single is a significant risk factor for suicide among women [9]. In fact, studies from Asian countries support the findings of our results. Lal and Sethi report in a hospital-based study that most of the women who commit suicide in India are married and under the age of 30 years [84]. Another case-control study from China claims that married women are more vulnerable to suicidality [90].

In South-East Asia, morbidity and mortality by suicide among women peak between the ages of 15 and 29 years, which also corresponds to the traditional age for female marriage in this region [12]. One possible explanation for the comparatively higher ratio of female-to-male suicides found in South-East Asian countries compared to those in Western countries, especially among young married women, could be cultural attitudes toward the woman’s traditional role in marriage [16]. Most South-East Asian societies are highly patriarchal, and due to several social and cultural factors, marital status plays an important role in the lives of women. In the countries of this region, where arranged and even forced marriages are quite frequent, social and cultural pressure to remain committed to unacceptable and abusive marriages can be one of the contributors to elevated suicidality among women [23].

Consistent with several previous studies, the findings of this review indicate that suicidality among women in the WHO South-East Asia Region is positively associated with low or no education, unemployment, low socioeconomic position, and poverty [84,91,92,93,94,95]. Lack of economic independence among South-East Asian women might lead to psychosocial stresses, which could further contribute to increased suicidality. Moreover, the findings support prior research indicating that social, cultural, or religious pressure on women might discourage them from attaining higher education, employment, high socioeconomic position, and economic independence [61,96,97,98]. Consequently, many South-East Asian women may be forced by the circumstances to remain entrapped within unhappy marriages and financially dependent arrangements. This might leave them in the long term with no other choice than committing suicide as a means of escape or a “permanent solution of their temporary problems”.

The predominance of rural women among the individuals who attempt or commit suicide is in agreement with some previous studies from the South-East Asia Region [62,78,80,91]. The higher rate of suicidality among rural women in South-East Asia may be explained by rapid migration, financial hardship, lack of economic opportunities, isolation, absence of social support, easy accessibility to lethal pesticides, and limited access to quality health care [91,99]. Likewise, consistent with previous studies, the most common method of suicide among women is by ingestion of pesticides [100,101,102,103,104,105]. These lethal pesticides are commonly used by rural farming communities, thus facilitating easy access, and contributing to elevated suicidality among rural women. Choudhury et al. also support these findings and reveal that more than 65% of rural women who attempt suicide in India consume pesticides [106].

The results confirm the well-established strong association between domestic abuse, intimate partner violence, and suicide among women. Research from around the globe also suggests that physical, emotional, and sexual abuse increases the risk of attempted suicide and suicide death among women [107,108,109,110,111]. However, the literature considered in this review highlights that the contribution of gender disadvantage, domestic violence, and sexual abuse to female suicidality in South-East Asia is much more predominant as compared to other parts of the world. For instance, a study from India outlines that domestic violence is the main reason for female suicide in 36% of the cases [44].

The fatal combination of regular abuse, feeling of powerlessness, lack of agency, and subsequent shame faced by South-East Asian women may force them to commit suicide. Consequently, wife abuse remains one of the most significant predictors of suicide among women in this region. According to Counts, case studies from several LMICs illustrate that if a woman’s support group does not defend her when she is the victim of violence that passes the bounds of normative behavior, her suicide may be revenge suicide, intended to force others to take vengeance on the abusive husband [107]. Another likely explanation could also be the linkage of domestic violence to the development of mental illness in women. Several previous studies support this association and show that intimate partner violence and especially sexual abuse is a strong predictor of depression and other common mental disorders [112,113,114,115,116]. Depression could play an intermediary role in the relationship between intimate partner violence and suicide in women. There is a probability that the strong association between female suicidality and domestic violence is confounded or mediated by common mental disorders [117].

The findings related to the association between husband’s harmful use of alcohol and increased risk of suicide among wives are also consistent with previous research [10,118]. Other studies from the region confirm these findings and highlight that many women commit suicide due to the alcohol use disorder of their husbands [10].

The publications considered for this review identify marital and family conflicts as significant predictors of attempted suicide and suicide among females. These findings support the results of several other previous studies from the region, which also mention family conflict as a main predisposing factor for suicide among women [61,62,63,64,65,66,67]. The Indian National Crime Record Bureau verifies that in almost 23% of registered cases, family conflict is the major reason leading to suicide among women below 30 years old [62]. Several social and cultural factors can cause marital and family conflicts. In Bangladesh, India, and Nepal, a male child is highly preferred and there is huge pressure on women to give birth to male offspring. Giving birth to a female child could be a reason for conflict with the husband and in-laws. Likewise, female infertility could also be a cause of constant intra-household disputes and may contribute to female suicidality. Our findings highlight the association between childlessness and increased risk of suicide among South-East Asian women. This association could be mediated through family disputes leading to marital disharmony, harassment, domestic violence, reduced self-esteem, lower social status, and mental health issues. The evidence in this manuscript about the relationship between childlessness and female suicide is consistent with previous research from other developing countries [62,68,69]. Extramarital sexual relationships of the husband may also contribute to family disputes and elevated risk of suicide among wives.

### 4.1. Limitations

From the research perspective, this scoping literature analysis has a few limitations. Only literature published in the English language between 1990 and May 2021 was considered. During the screening and selection process, it was obvious that evidence on female suicidality and related risk factors in South-East Asia is extremely scarce. As suicide is a very sensitive issue in the South-East Asia Region, the possibility of underreporting and misclassification of attempted suicide and suicide deaths among women due to stigma cannot be ruled out. Due to the nature of the scoping review, we also did not assess for methodological quality. Factors such as the varying suicide registration systems used by different countries in this region add to the challenges of conducting research on this public health issue. Consequently, limited relevant information was retrieved on female suicide in countries of the WHO South-East Asia Region. Most of the publications found were from India. Not a single article was found focusing on suicide from Bhutan, Indonesia, North Korea, Maldives, Myanmar, or Timor-Leste. The lack of publications from these countries and unequal representation of the data might have undermined additional risk factors responsible for attempted suicide and suicide death among women in this region.

Some studies included in the review are based on self-reports or verbal autopsies from the family members, which may have resulted in the overreporting or underreporting of some risk factors due to recall bias or lack of voluntary disclosure. It is possible that some of the associations demonstrated may be a consequence of socially desirable responses. For the risk factors for attempted suicides, another limitation is the inability to conclude causality and/or direction of the relationship, as many reviewed studies had a cross-sectional study design. Recall bias is also a problem in the studies relying on the reports of lifetime occurrence of domestic violence and mental health issues. Furthermore, there is a probability that in South-East Asian countries mental illness is underestimated as a risk factor for suicide because of the stigma associated with mental disorders. Due to the above-mentioned limitations, all findings should be interpreted with caution.

### 4.2. Policy Recommendations and Implications for Suicide Prevention

The present literature analysis shows that a complex interaction of several causative factors and the sociocultural context contributes to female suicidality in South-East Asia. Even though there are some similarities with the factors responsible for female suicidality in high-income countries, our findings outline that the pattern and causation of suicides among women in this region are much different. Considering the enormity of the problem, it is important to recognize female suicides as a public health problem and evolve culture-specific preventive measures in each country of this region. In South-East Asia, treatment of mental illnesses is certainly not enough to reduce female suicidality without solving the underlying structural, sociocultural, and socioeconomic problems [8]. Therefore, suicide prevention strategies must be multi-pronged and gender-specific and should combine strategies to reduce gender disadvantage and domestic violence. Furthermore, providing poverty relief funds and strengthening the health system in the early diagnosis and treatment of common mental disorders must be an integral part of suicide prevention strategies [8].

Most of the countries in the region have not yet established a national suicide-prevention strategy, with the exception of Bhutan, Sri Lanka, and Thailand. Thus, all countries in South-East Asia must develop a comprehensive and gender-specific national suicide-prevention strategy and also should allocate sufficient human and financial resources for it. In addition, the establishment of good monitoring and reporting systems that facilitate the collection of reliable data on the prevalence, demographic patterns, methods, and associated risk factors of both attempted suicide and suicide death is necessary [12]. Accordingly, further future research and a better understanding of the association between major suicide-related risk factors and suicidal behavior among women in the WHO South-East Asia Region is inevitable to inform and improve the region’s public health suicide prevention programs [5].

Since underlying reasons for suicides are multifactorial, it is important that prevention strategies must include both macro- and micro-level initiatives focusing on individual, family, and societal levels [44]. Socioculturally relevant strategies must be incorporated into health, education, and welfare programs in all the countries of the region. At the country level, policymakers and politicians must prioritize the optimal use of resources to decrease the gender gap in terms of literacy and employment, and reduce socioeconomic inequalities, especially in rural areas. Restricting the easy availability and accessibility to highly lethal pesticides in the South-East Asia Region should also be a top priority in suicide prevention, as it is already proven to be an effective strategy [119]. The establishment or improvement of quality emergency care in rural areas of the region could be another beneficial strategy to save human lives especially due to pesticide ingestion [44].

Several micro-level initiatives at the individual and family level are also necessary. These initiatives and interventions should aim at improving skills to recognize and deal with crisis situations, strengthening interpersonal problem-solving skills, and promoting help-seeking behavior. Furthermore, community-based intervention strategies should be developed to tackle issues such as marital and intergenerational conflicts and to strengthen family interactions [12].

### 4.3. Future Research

Considering the magnitude of the problem, identification of specific socioeconomic, sociocultural, and psychological stressors that lead to an increased risk of suicide among South-East Asian women is necessary [12]. However, this review highlights the lack of extensive knowledge on suicide in most countries of the South-East Asia Region and the urgency to better understand this complex public health phenomenon. The situation is further compounded by the fact that there is a lack of women-specific research on suicide in this region. There is an urgent need for research in the countries of Bhutan, Indonesia, North Korea, Maldives, Myanmar, and Timor-Leste. To enhance the current state of knowledge, further research should be preferably conducted by local scholars familiar with local languages, customs, and culture [46]. As gender disadvantage plays a significant role in the region, research among the victims of psychological, physical, or sexual violence can provide some insight into circumstances and triggers of this violence. Among survivors of such violence, police reporting, help-seeking, and healthcare-seeking behaviors and related obstacles should also be studied [48]. More research is required to recognize strategies and interventions that are appropriate, feasible, and effective to reduce or prevent female suicidality [12]. Coping strategies used by female suicide attempters in the region should be studied to improve coping and resilience interventions [31].

Efforts are needed to assess and analyze existing reporting and registration systems, surveillance systems, and data availability in each country of the region. One area of research that requires particular attention is that of the laws regarding suicide and attempted suicide in the countries of this region [16]. The “criminalization” of suicidal behavior has led to stigma, avoidance of seeking help, and a lack of interest of professionals in developing suicide preventive programs [120]. Therefore, comprehensive research about the impacts of criminalization of attempted suicide and outcomes of persecution of attempters by the police in the South-East Asia Region is essential. This study emphasizes the immediate need for actions from all stakeholders at the regional, national, and international levels; otherwise, women in this region will keep committing suicide as currently, it is the only permanent solution to every pain and problem of life.

## 5. Conclusions

This scoping review highlights an important public health problem in South-East Asia, attempted suicide, and suicide death in girls and women. Suicide is a consequence of a complex interplay of multiple socioeconomic, psychological, and cultural factors. Among all these, gender disadvantage plays a crucial role in increasing the vulnerability of girls and women in this region. Low education, rural residence, low socioeconomic position, poverty, limited exposure to the world outside one’s home, and marital conflicts are some of the key determinants that need to be considered while designing, implementing, and evaluating public health interventions to prevent suicide in South Asia.

## Data Availability

All data used in this study are publicly available.

## Supplementary Materials

The following supporting information can be downloaded at: https://www.mdpi.com/article/10.3390/ijerph21121658/s1, Table S1: Risk factors for suicide and attempted suicide among women.

## Appendix A

Additional file 1: Search strategy

Identification of articles

Published studies, reports, and reviews were searched through a systematic search using the following strategies. To identify suitable peer-reviewed and individual publications, the databases PubMed, MEDLINE, EBSCOhost, PsycINFO, and EMBASE were searched. Searches were carried out by using the following search terms: [suicid* AND women AND South-East Asia] OR [suicid* AND women AND WHO South-East Asia region] OR [suicid* AND women AND WHO SEAR] OR [suicid* AND women AND India] OR [suicid* AND women AND Bangladesh] OR [suicid* AND women AND Bhutan] OR [suicid* AND women AND Nepal] OR [suicid* AND women AND North Korea] OR [suicid* AND women AND Sri Lanka] OR [suicid* AND women AND Indonesia] OR [suicid* AND women AND Maldives] OR [suicid* AND women AND Myanmar] OR [suicid* AND women AND Thailand] OR [suicid* AND women AND Timor-Leste]. The initial identification of relevant publications was based on title, keywords, and abstracts. The titles and abstracts of papers identified through the database search were screened. These abstracts were examined and the full text of papers that reported on the predictors or risk factors of attempted or suicide death among women in the countries of South-East Asia were retrieved. These full texts were then assessed to determine their compliance with the eligibility criteria mentioned below. To identify additional articles, a manual search was performed, based on the bibliographies of the published studies (“snowballing”) on risk factors for female suicide.

## Appendix B

**Table A1:** Preferred Reporting Items for Systematic reviews and Meta-Analyses extension for Scoping Reviews (PRISMA-ScR) Checklist.

Section	Item	Prisma-scr Checklist Item	Reported on Page #
Title			
Title	1	Identify the report as a scoping review.	Page 1
Abstract			
Structured summary	2	Provide a structured summary that includes (as applicable): background, objectives, eligibility criteria, sources of evidence, charting methods, results, and conclusions that relate to the review questions and objectives.	Page 2
Introduction			
Rationale	3	Describe the rationale for the review in the context of what is already known. Explain why the review questions/objectives lend themselves to a scoping review approach.	Page 3/4
Objectives	4	Provide an explicit statement of the questions and objectives being addressed with reference to their key elements (e.g., population or participants, concepts, and context) or other relevant key elements used to conceptualize the review questions and/or objectives.	Page 3/4
Methonds			
Protocol and registration	5	Indicate whether a review protocol exists; state if and where it can be accessed (e.g., a Web address); and if available, provide registration information, including the registration number.	Page 4
Eligibility criteria	6	Specify characteristics of the sources of evidence used as eligibility criteria (e.g., years considered, language, and publication status), and provide a rationale.	Page 4/5
Information sources *	7	Describe all information sources in the search (e.g., databases with dates of coverage and contact with authors to identify additional sources), as well as the date the most recent search was executed.	Page 4
Search	8	Present the full electronic search strategy for at least 1 database, including any limits used, such that it could be repeated.	Page 4/Appendix 1
Selection of sources of evidence †	9	State the process for selecting sources of evidence (i.e., screening and eligibility) included in the scoping review.	Page 4/5
Data charting process ‡	10	Describe the methods of charting data from the included sources of evidence (e.g., calibrated forms or forms that have been tested by the team before their use, and whether data charting was performed independently or in duplicate) and any processes for obtaining and confirming data from investigators.	Page 5
Data items	11	List and define all variables for which data were sought and any assumptions and simplifications made.	Page 5/6
Critical appraisal of individual sources of evidence §	12	If done, provide a rationale for conducting a critical appraisal of included sources of evidence; describe the methods used and how this information was used in any data synthesis (if appropriate).	Page 5/6
Synthesis of results	13	Describe the methods of handling and summarizing the data that were charted.	Page 5
Results			
Selection of sources of evidence	14	Give numbers of sources of evidence screened, assessed for eligibility, and included in the review, with reasons for exclusions at each stage, ideally using a flow diagram.	Page 6
Characteristics of sources of evidence	15	For each source of evidence, present characteristics for which data were charted and provide the citations.	Page 6
Critical appraisal within sources of evidence	16	If done, present data on critical appraisal of included sources of evidence (see item 12).	-
Results of individual sources of evidence	17	For each included source of evidence, present the relevant data that were charted that relate to the review questions and objectives.	Table S1
Synthesis of results	18	Summarize and/or present the charting results as they relate to the review questions and objectives.	Table S1
Discussion			
Summary of evidence	19	Summarize the main results (including an overview of concepts, themes, and types of evidence available), link to the review questions and objectives, and consider the relevance to key groups.	Page 11–16
Limitations	20	Discuss the limitations of the scoping review process.	Page 16, 17
Conclusions	21	Provide a general interpretation of the results with respect to the review questions and objectives, as well as potential implications and/or next steps.	Page 20
Funding			
Funding	22	Describe sources of funding for the included sources of evidence, as well as sources of funding for the scoping review. Describe the role of the funders of the scoping review.	Page 20, 21

JBI = Joanna Briggs Institute; PRISMA-ScR = Preferred Reporting Items for Systematic Reviews and Meta-Analyses extension for Scoping Reviews. * Where *sources of evidence* (see second footnote) are compiled from, such as bibliographic databases, social media platforms, and Web sites. † A more inclusive/heterogeneous term used to account for the different types of evidence or data sources (e.g., quantitative and/or qualitative research, expert opinion, and policy documents) that may be eligible in a scoping review as opposed to only studies. This is not to be confused with *information sources* (see first footnote). ‡ The frameworks by Arksey and O’Malley (6) and Levac and colleagues (7) and the JBI guidance (4, 5) refer to the process of data extraction in a scoping review as data charting. § The process of systematically examining research evidence to assess its validity, results, and relevance before using it to inform a decision. This term is used for items 12 and 19 instead of “risk of bias” (which is more applicable to systematic reviews of interventions) to include and acknowledge the various sources of evidence that may be used in a scoping review (e.g., quantitative and/or qualitative research, expert opinion, and policy document). From: Tricco AC, Lillie E, Zarin W, O’Brien KK, Colquhoun H, Levac D, et al. PRISMA Extension for Scoping Reviews (PRISMAScR): Checklist and Explanation. *Ann. Intern. Med.*, 2018.
