# Supplementary material for: Risk Factors for Attempted Suicide and Suicide Death Among South-East Asian Women: A Scoping Review
Source: Int J Environ Res Public Health. 2024 Dec 12;21(12):1658. doi: 10.3390/ijerph21121658 (PMC11675859; doi:10.3390/ijerph21121658)
Supplement: Supplementary file 1 [file ijerph-21-01658-s001.zip › ijerph-3216337-supplementary.pdf]

Author / Year	Title	Risk factors for female suicide	Type
Ahmed et al. (2004)	Violent deaths among women of reproductive age in rural Bangladesh	Oppression, intimate partner violence, young age, gender disadvantage, unmarried, divorced, widowed, poverty, childlessness, rejection of marriage offers, forced marriages, pre-martial affairs, illegitimate pregnancies, dowry	Study
Amudhan et al. (2020)	A population-based analysis of suicidality and its correlates: findings from the National Mental Health Survey of India, 2015-16	Lower educational level, residing in urban metropolitan cities, widowed, separated, divorced, unemployed, belonging to the lowest income quintile, depressive disorders	Study
Arafat (2016)	Suicide in Bangladesh: A mini review	Low socioeconomic position, lower educational level, married, younger age, family conflict, gender disadvantage, lack of economic opportunities, early marriage, joint families, arranged marriage, chronic illness, marital conflicts	Review
Babu & Babu (2011)	Dowry deaths: a neglected public health issue in India	Dowry	Study
Babu et al. (2008)	Prevalence and correlates of suicidality among Indian women with post-partum psychosis in an inpatient setting	Depressive symptoms in post-partum psychosis, post-partum psychiatric disorder	Study
Bagley et al. (2017)	"High Rates of Suicide and Violence in the Lives of Girls and Young Women in Bangladesh: Issues for Feminist Intervention"	Sexual violence, younger age, poverty, lower educational level, dowry, domestic violence, lower socioeconomic position, early marriage, gender disadvantage, family conflict, poverty, infertility, marital conflicts	Review
Batra (2003)	Burn mortality: recent trends and sociocultural determinants in rural India	Torture by in-laws, dowry, chronic illness, financial problems, family conflicts, extramarital sexual relations of the husband, failing to produce children	Study
Chowdhary & Patel (2008)	The effect of spousal violence on women's health: Findings from the Stree Arogya Shodh in Goa, India	Domestic violence, intimate partner violence	Study
Cousins (2016)	Nepal's silent epidemic of suicide	Mental health issues, family conflicts, young age, female gender, poverty, lack of economic opportunities, arranged marriages, gender-based violence, human trafficking	Review
Devries et al. (2011)	Violence against women is strongly associated with suicide attempts: evidence from the WHO multi-country study on women's health and domestic violence against women	Domestic violence, intimate partner violence, sexual violence, childhood sexual abuse, having a mother who had experiences intimate partner violence	Study

Ebenezer & Joge (2016)	Suicide in Rural Central India: Profile of Attempters of Deliberate Self Harm Presenting to Padhar Hospital in Madhya Pradesh	Interpersonal conflicts, mental illness, alcohol use disorder	Study
Ellsberg et al. (2008)	Intimate partner violence and women's physical and mental health in the WHO multi-country study on women's health and domestic violence: an observational study	Domestic violence, intimate partner violence	Study
Feroz et al. (2012)	A Community Survey on the Prevalence of Suicidal Attempts and Deaths in a Selected Rural Area of Bangladesh	Younger age, living in rural areas, married, unemployment, lower socioeconomic position, spousal violence, family conflicts, family history of suicidal behaviour, chronic physical illness	Study
Gupta et al. (2015)	Intra-household evaluations of alcohol abuse in men with depression and suicide in women: A cross-sectional community-based study in Chennai, India	Living in a household with alcoholics	Study
Gururaj et al. (2004)	Risk factors for completed suicides: a case-control study from Bangalore, India	Prior suicide attempts, family history of suicidal behaviour, mental illness, physical illness, domestic violence, emotional abuse, alcohol use disorder in husband, family conflict, marital conflict, unemployment, financial problems, lower educational level	Study
Hagaman et al. (2018)	Suicide in Nepal: Qualitative Findings from a Modified Case-Series Psychological Autopsy Investigation of Suicide Deaths	Lack of education, poverty, violence, intimate partner violence, family conflicts, family history of suicidal behaviour, mental illness, interpersonal conflicts, prior suicide attempts	Study
Indu et al. (2020)	Domestic Violence as a Risk Factor for Attempted Suicide in Married Women	Domestic violence, younger age, poor social support, having a family history of psychiatric disorders, substance use disorders, wives of alcoholics, middle socioeconomic group	Study
Jain et al. (1999)	A study of hopelessness, suicidal intent and depression in cases of attempted suicide	Younger age, unemployment, easy access to pesticides, financial problems, educational stress, family conflicts, family history of suicidal behaviour, common mental disorders, depression	Study
Khan (2002)	Suicide on the Indian subcontinent	Interpersonal conflicts, marital status, family conflicts, domestic violence	Review
Khan et al. (2005)	Psychological autopsy of suicide - a cross-sectional study	Younger age, financial problems, educational stress, failing to produce children, family conflicts, wife of an alcoholic, mental illness, family history of suicidal behaviour, common mental disorders	Study

Knipe et al. (2017)	Is socioeconomic position associated with risk of attempted suicide in rural Sri Lanka? A cross-sectional study of 165 000 individuals	Lower socioeconomic position, lower level of education	Study
Knipe et al. (2018)	Attempted suicide in Sri Lanka - An epidemiological study of household and community factors	Low socioeconomic position, women living in households with alcoholics	Study
Knipe et al. (2019)	Socioeconomic position and suicidal behaviour in rural Sri Lanka: a prospective cohort study of 168,000+ people	Lower socioeconomic position, lower level of education	Study
Konradsen et al. (2005)	"Reaching for the bottle of pesticide—A cry for help. Self-inflicted poisonings in Sri Lanka"	Arranged marriage, domestic violence, sexual violence, living in a household with alcoholics, young age, forced marriage, marital conflicts, illegitimate pregnancies, extramarital affair	Study
Kumar (2003)	Burnt wives--a study of suicides	Dowry, joint families, young age	Study
Kumar (2004)	Poisoning deaths in married women	Dowry	Study
Kumar et al. (2006)	Gender differences in medically serious suicide attempts: a study from south India	Lower educational level, unemployment, from rural areas, psychiatric disorders	Study
Li et al. (2021)	Suicide attempt and its associated factors amongst women who were pregnant as adolescents in Bangladesh: a cross-sectional study	Adolescent pregnancy	Study
Manohar & Kannappan (2010)	Domestic Violence and Suicidal Risk in the Wives of Alcoholics and Non-alcoholics	Domestic violence, wives of alcoholics	Study
Marahatta et al. (2017)	Suicide burden and prevention in Nepal: The need for a national strategy	Younger age, mental disorder, female gender, interpersonal conflicts, family conflicts, family history of attempted suicide, substance-use disorders, easy access to pesticides	Review
Marecek (2006)	Young Women's Suicides In Sri Lanka: Cultural, Ecological and Psychological Factors	Family conflicts, domestic violence, sexual violence, alcohol abuse	Review
Maselko & Patel (2008)	Why women attempt suicide: the role of mental illness and social disadvantage in a community cohort study in India	Mental illness, common mental disorders, physical illness, gender disadvantage, domestic violence, poverty, having family debt, young age at marriage	Study
Narang et al. (2000)	Attempted suicide in Ludhiana	Low education level, younger age, married	Study
Panyayong et al. (2018)	Psychiatric disorders associated with intimate partner violence and sexual	Intimate partner violence, sexual violence	Study

	violence in Thai women: A result from the Thai National Mental Health Survey		
Parkar et al. (2008)	Gender, suicide, and the sociocultural context of deliberate self-harm in an urban general hospital in Mumbai, India	Domestic strife, family conflicts, financial problems, living in a household with alcoholics, sterility	Study
Peltzer & Pengpid (2017)	Associations between intimate partner violence, depression, and suicidal behavior among women attending antenatal and general outpatients hospital services in Thailand	Intimate partner violence	Study
Pengpid et al. (2018)	Intimate partner sexual violence and risk for femicide, suicidality and substance use among women in antenatal care and general out-patients in Thailand	Intimate partner violence, sexual violence	Study
Pillai et al. (2009)	Violence, psychological distress and the risk of suicidal behaviour in young people in India	Common mental disorders, gender disadvantage, domestic violence, sexual violence, making independent decisions, lack of education, female gender, premarital sex, living in rural areas	Study
Reza et al. (2013)	Risk Factors of Suicide and Para Suicide in Rural Bangladesh	Younger age, living in rural areas, married, lower socioeconomic position, family conflicts, family history of suicidal behaviour, chronic physical illness, financial problems, dowry disputes, mental illness	Study
Sabri et al. (2015)	Motives and characteristics of domestic violence homicides and suicides among women in India	Domestic violence, dowry, intimate partner violence	Review
Shahmanesh et al. (2009)	Suicidal behavior among female sex workers in Goa, India: the silent epidemic	Gender disadvantage, gender-based violence, sex work, social disadvantage, intimate partner violence, sexual violence	Study
Sharma et al. (2005)	Dowry--a deep-rooted cause of violence against women in India	Dowry, socioeconomic status	Study
Shukla, Verma et al. (1990)	Suicide in Jhansi City	Younger age, married, domestic strife, low education level, financial problems, chronic physical illness, mental illness, gender disadvantage, dowry disputes	Study
Singh et al. (2021)	Fertility, economic development, and suicides among women in India	Inadequate female autonomy	Study
Srivastava & Kumar (2005)	Suicidal ideation and attempts in patients with major depression: Sociodemographic and clinical variables	Younger age, married, unemployment, living in urban areas, failing to produce sons, common mental disorders, depression	Study

Srivastava et al. (2004)	Risk factors associated with attempted suicide : a case control study	Younger age, low educational level, unemployment, financial problems, adolescent pregnancy, family conflicts, mental illness, chronic physical illness,	Study
Thapaliya et al. (2018)	Suicide and self harm in Nepal: A scoping review	Interpersonal conflicts, marital conflicts, socioeconomic problems, mental illness, female gender, domestic strife, living in a household with alcoholics, educational stress, low socioeconomic status, chronic physical illness, failure in romantic relationships, forced marriage, inter-caste marriage	Review
Vijayakumar (2010)	Indian research on suicide	Physical illness, mental illness, idiopathic pain, interpersonal conflicts, financial problems, extramarital affair, domestic strife, lower educational level, joint family, unemployment, stressful live event in the last six month	Review
Vijayakumar (2015)	Suicide in women	Young age, female gender, postpartum psychosis, depression, abortion, fertility problems, intimate partner violence, domestic violence, dowry, childhood sexual abuse, young age at marriage	Review
Vijayakumar (2017)	Challenges and opportunities in suicide prevention in South-East Asia	Easy access to pesticides, arranged marriage, forced marriage, disapproved relationship by families, unrequited love, domestic violence, wives of alcoholics, dowry, failing to produce a son, infertility	Review
Vijayakumar et al. (2021)	A descriptive mapping review of suicide in vulnerable populations in low and middle countries	Early marriage, dowry, failing to produce children, gender-based violence, family conflicts, lower educational level, sexual violence, patriarchal restrictions on their space, choices and means of self-expression	Review
Vishnuvardhan & Saddichha (2012)	Psychiatric comorbidity and gender differences among suicide attempters in Bangalore, India	Family conflicts, physical illness, financial problems, chronic illness, psychiatric disorders, lower educational level, unmarried	Study
